# Culturomics from field-grown crop plants using dilution to extinction, two-step library preparation and amplicon sequencing

**DOI:** 10.1099/mic.0.001571

**Published:** 2025-06-17

**Authors:** Eglantina Lopez-Echartea, Nicholas Dusek, Mallory Misialek, Mohammad Al Mahmud-Un-Nabi, Riley Williamson, Komal Marathe, Barney A. Geddes

**Affiliations:** 1 Department of Microbiological Sciences, North Dakota State University, Fargo, ND, USA; 2 Center for Computationally Assisted Science and Technology, North Dakota State University, Fargo, ND, USA

**Keywords:** culturomics, microbial ecology, microbiome, synthetic community

## Abstract

Culturomics approaches have advanced microbial research by enabling the high-throughput isolation and characterization of a broader range of bacterial taxa, including some previously considered unculturable. Here, we present the testing and optimization of a protocol for isolating and identifying hundreds of cultivable microbes from field-grown plants. This protocol was tested and optimized using the root microbiomes of field-grown corn and pea plants under varying environmental conditions in ND, USA. By employing dilution-to-extinction culturing and a two-step barcoding PCR strategy targeting the V4 region of the 16S rRNA gene, we identified over 200 unique bacterial isolates. The optimized bioinformatic pipeline, built around the DADA2 package, ensured accurate amplicon sequence variant detection and taxonomy assignment. The resulting bacterial isolates span diverse phylogenetic groups, including plant-associated taxa known for promoting plant growth and mitigating stress. Our findings highlight the value of culturomics in generating microbial collections for synthetic community design and advancing plant–microbe interaction research. The protocol’s scalability, cost-effectiveness and robust performance demonstrate its potential for widespread application in agricultural microbiome studies.

## Data Availability

Sequencing data was deposited in the NCBI SRA under the BioProject PRJNA1220177, with accession numbers listed in Table S1, available in the online Supplementary Material.

R scripts used for data analysis are available at https://github.com/NDSU-Geddes-Lab/cultured-microbe-identification.

Protocols for automated library preparation have been published on protocols.io and are available at DOI: dx.doi.org/10.17504/protocols.io.eq2ly662egx9/v1.

**Impact Statement**

High-throughput isolation and characterization of cultivable microbes from plant microbiomes are crucial for advancing microbiome research. However, efficiently recovering a diverse range of bacterial taxa remains a challenge due to high costs and labour-intensive protocols. Our optimized culturomics protocol integrates dilution-to-extinction culturing with a two-step barcoding PCR strategy, enhancing recovery rates and reducing costs whilst maintaining high accuracy. By employing next-generation sequencing and a streamlined bioinformatic pipeline built around the robust DADA2 workflow for amplicon sequencing, the method enables the scalable recovery of hundreds of unique bacterial isolates. This approach makes significant advancements over traditional culturing methods and other high-throughput cultivation protocols, providing an efficient, cost-effective platform for generating microbial collections essential for synthetic communities, comparative genomics, and agricultural microbiome applications.

## Introduction

The first isolates of pure bacterial cultures were introduced in the late nineteenth century by traditional solid culture media [1]. Although this technique has been one of the pillars of microbiology as a research field, advances in culturing microbes have not kept pace in recent years with advances in our ability to characterize microbial communities by next-generation sequencing (NGS) [2,3]. However, in this multi-omics era where thousands of terabytes of sequence data are generated year after year, cultivation is still irreplaceable.

Microbial cultures are necessary for the evaluation of metagenomics data, the assessment and characterization of genes and genomes, the isolation of bacterial and archaeal viruses and testing hypotheses [4]. Moreover, the characterization and isolation of new bacterial isolates are of high relevance due to their multiple applications in biotechnology, bioremediation, bioactive compounds and bioprospection [5,9]. The field of agriculture will also benefit from the high-throughput cultivation (HTPC) and fast identification of microbes as the plant microbiome could provide protection against pathogens, fix nitrogen, promote plant growth and decrease stress from abiotic factors such as drought, salinity and contaminants [10,14]. Combining cultured microbes into synthetic bacterial communities (SynComs) has also gained significant traction in recent years to facilitate a reductionist, ‘bottom-up’ approach to the study of microbial ecology and complement the wealth of ‘top-down’ amplicon sequencing studies [15,17].

Microbes represent the largest set of biomass on Earth and are represented in all domains of life, yet 90% of their diversity is still unexplored [18], in part because most of the prokaryotic taxa have not been yet cultured [4]. The reason for this uncultivability relies on several factors such as the absence of factors produced by other microbes [19,20], interspecies interactions such as symbiotic growth [21,22] and variations in the growth rate [23] and dormancy [24] of individual microbes. Several groups have attempted to increase culturability through incorporating radically altered approaches and technologies such as microfluidic droplets [25], microcapsules [26] and miniature diffusion chambers that are placed in the environment [27]. Still, perhaps the most significant advances in microbial culturing have resulted from more incremental improvements on traditional culturing methods by optimizing culture conditions. For example, the improvement in media, nutrient addition and approaches like ‘dilution-to-extinction’ have made great advances in fields like marine microbiology where microbes are slow growers with low nutrient requirements [3,28, 29]. Pioneered in the human gut microbiome, cutting-edge approaches dubbed ‘culturomics’ involve combining optimized growth conditions with high-throughput workflows enabled by robotics, automation and next-generation sequencing approaches for the identification of target microbes of interest [30,33].

Culturomics approaches have been applied to the plant microbiome and shown great success where the proportion of cultivatable taxa has been found to be relatively high [34,35]. In this study, we further advance culturomics efforts in the plant microbiome, by (1) testing the dilution-to-extinction approach for cultivation of microbes from field-grown corn and pea plants in ND, USA, and (2) developing a two-step PCR and bioinformatic strategy for identification of cultured microbes. We combined these tests into an updated protocol for culturomics from the plant microbiome.

## Methods

### Sampling and sample preparation

Pea plants were collected in July 2022 from a *Fusarium*/*Aphanomyces* root rot trial in Williston, ND, and a salinity trial in Carrington, ND. Corn plants were collected in July 2023 from a field trial in Gardner, ND, that included variable nitrogen fertilizer application rates [0 or 200 lbs ac^−1^ (~224 kg ha^−1^)] at planting. Samples were collected from field locations, leaving the rhizosphere intact and transported on ice to the laboratory. Samples were processed in the laboratory by removing loosely associated microbes with a series of washes in PBS, followed by cutting and grinding of plant tissues into a slurry, as previously described [34]. An aliquot of the slurry was separated for microbial community composition analysis, and the remaining was used for culturing.

### Bacterial culturing

A slurry from each plant root sample was used for culturing by dilution to extinction in 10% tryptic soy broth (TSB) as previously described [34] and included a threefold serial dilution from an initial 2000× dilution of the plant slurry to 486,000×. A total of 20 plates for each dilution were inoculated for each plant sample. Culture plates were incubated for 12 days at room temperature. Individual 96-well plates from dilution-to-extinction culturing were then selected based on the proportion of growth in wells (18–55%). An aliquot from each well was transferred to a new 96-well plate for cultured bacteria identification, and the remaining contents were combined with glycerol (40% final volume) and stored at −80 °C.

For the purification of isolates, streak plating from single colonies was used on TSB agar plates. Following three rounds of purification, isolates were resuspended in TSB and combined with glycerol (40% final volume) for long-term storage.

### PCR amplification for bacterial identification

Genomic DNA was extracted from bacterial culture aliquots using an alkaline lysis method. Amplification of the bacterial 16S rRNA gene was performed using an adaptation to the standard Illumina two-step PCR approach [36]. Specifically, we incorporated four to six nucleotide barcodes into 12 unique forward and 8 unique reverse primers targeting the V4 region of the 16S rRNA gene (V4_515F and V4_805R) in between the V4 binding site and adaptor tail binding sites for secondary amplification (Table S2). These primers were used with a unique forward primer for each column of the 96-well plate and each reverse primer for each row such that every well would receive a unique primer combination. The primary PCR amplification used KAPA HotStart polymerase (Kapa Biosystems, USA), with cycling conditions of an initial denaturation at 95 °C for 3 min, followed by 26 cycles of 95 °C for 30 s, 55 °C for 30 s and 72 °C for 30 s, with a final extension at 72 °C for 5 min. PCR products from each of the 96 wells were then pooled together and purified using Mag-Bind beads (Omega Bio-tek, USA). A second round of PCR was performed on each pooled plate sample from PCR1, incorporating Nextera primers with sequencing adapters [34]. This was done under conditions similar to the primary PCR, but the number of cycles was reduced from 26 to 9. The PCR products were again purified using Mag-Bind beads (Omega Bio-tek, USA), followed by quantification using a Quant-iT PicoGreen dsDNA Assay Kit (Thermo Fisher Scientific, USA). Libraries were pooled in equimolar amounts and sequenced on an Illumina MiSeq platform at the Core Lab of the Department of Microbiological Sciences of the North Dakota State University following standard protocols.

PCR for root slurry microbiome profiling was performed using the same reagents and cycling conditions as described for bacterial isolate amplification. The only difference was that the PCR1 primers (V4_515F and V4_R, Table S2) did not include the four to six nucleotide barcodes used in the HTPC workflow.

PCR amplification and Sanger sequencing were used to validate the bacterial 16S rRNA gene sequences from purified isolates. The full-length 16S rRNA gene was amplified using 27F and 1492R primers, following previous PCR1 conditions but with an extended annealing time of 1 min. PCR products were treated with ExoSAP-IT (Thermo Fisher Scientific, USA) and sequenced by Molecular Cloning Laboratories, USA, using Sanger sequencing.

### Bioinformatic analysis

FASTQ files from Illumina sequencing from the cultivation plates were demultiplexed using a custom script (microbeID.R from https://github.com/NDSU-Geddes-Lab/cultured-microbe-identification). Briefly, the script trims, merges and denoises reads using the DADA2 package [37]; demultiplexes sequences from each plate; and assigns them to wells based on the primer barcode combination and reported read number, purity and taxonomy of amplicon sequence variants (ASVs) in each well. The FASTQ files from Illumina sequencing from the root slurry were processed in the same way as in [38].

For the construction of the phylogenetic trees, V4 16S rRNA gene DNA sequences in FASTA format from the root slurry and the cultivation plates were aligned using multiple sequence alignment in R with the DECIPHER [39] and phangorn [40] packages. Briefly, the aligned sequences were used to compute a distance matrix using maximum likelihood, and an initial neighbour-joining tree was constructed. The tree was further refined using a maximum likelihood approach by fitting a general time reversible model. The optimized phylogenetic tree was saved as a Newick file and further processed in iTOL [41] to visualize and label the trees effectively.

To evaluate the ecological relevance of cultured isolates in Table S3, we first matched each isolate’s ASV sequence (ASV_V4) to the full set of ASVs detected in root slurry amplicon sequencing data for the corresponding plant system (pea or corn), using rarefied phyloseq objects as reference. For each matched ASV, we calculated its total abundance across all samples within that plant system and ranked it based on relative abundance. Isolates were then categorized into three groups: (1) core members, defined as ASVs present in at least 80% of samples and with >0.1% relative abundance (i.e. high occupancy and moderate-to-high abundance); (2) top 50%, defined as ASVs within the upper half of the relative abundance rank but not part of the core; and (3) rare, consisting of ASVs below the median in abundance. This classification allowed us to assess which isolates represent ecologically dominant or persistent members of the pea and corn microbiomes.

To identify core ASVs within each plant system, we first rarefied the sequencing depth of samples to a uniform value of 13,027 reads using the rarefy_even_depth() function in the phyloseq package [42]. Core ASVs were defined as those present in at least 70% of samples (prevalence threshold) within each plant type (pea or corn) and with a minimum relative abundance threshold of 0.1%. A custom function was used to convert the OTU table to relative abundance, binarize presence/absence data based on the detection threshold and calculate prevalence across samples.

To visualize differences in core ASVs across plant types, we generated bar plots and Venn diagrams using ggplot2 [43] and ggVennDiagram [44], based on phylum-level taxonomic classification and overlap between plant-specific core sets. To assess culturability and detection in HTPC datasets, we annotated each ASV by matching genus-level taxonomy to metadata on isolation status. Heatmaps were constructed using the pheatmap package [45], with samples collapsed by host type and ASV relative abundances aggregated at the genus level. Rows were annotated by culturability (based on isolate recovery) and HTPC detection status. These figures allowed us to evaluate which core taxa were successfully cultured, partially recovered or remained uncultured across plant hosts (see Figs S1 and S2).

## Results and discussion

### Microbe isolation from field-grown crop plants by dilution to extinction

We set out to test high-throughput culturomics in our experimental systems (field-grown crop plants with biotic and abiotic stress), starting with a previously published protocol that optimized the approach for root microbiome culturing from the plants [34]. Dilution to extinction relies on achieving a concentration where individual wells predominantly contain single cells, minimizing the competition between microbes and allowing for the growth of slower-growing or nutrient-limited species. This is done by selecting specific dilutions with a proportional growth in wells that indicates that most wells have been derived from a single viable bacterial cell according to a Poisson distribution model [34]. We tested a range of dilutions, from 2,000× to 486,000×, across different plant root samples (pea and corn) and environmental conditions (disease, salinity, fertilized and non-fertilized) collected from the field. Successful dilutions were identified based on growth rates in 96-well plates, with ideal dilutions defined as those yielding ~35% growth. At these levels, there is a high likelihood that each well contains either no cells or only a single viable cell.

For pea plants, we selected one dilution that closely matched these criteria (33% growth in the 54,000× dilution for peas growing in the disease-inoculated fields and 38% growth in the 18,000× dilution for peas growing in the salinity-induced fields). For corn plants, we selected the following dilutions: 18,000× dilution (55% growth) for corn plants growing in 200 lbs ac^−1^ (~224 kg ha^−1^) N fertilizer field and 6,000× and 18,000× dilutions (30% and 21% growth, respectively) for corn plants growing in the non-fertilized fields (Table 1). Overall, the dilutions within the range of the series recommended by Zhang *et al*. (up to 54,000×) were successful for our conditions, though given that in one case [34], only the maximum dilution was successful. Including further dilutions to 486,000× may be important for some plants with dense microbiome colonization such as those grown in rich field soil.

**Table 1.** Dilutions used for further culturing efforts and sequencing based on percentage of 96-well plate growth

Dilution	Pea (disease)	Pea (salinity)	Corn (0)	Corn (200)
2000×	TM	TM	TM	TM
6,000×	TM	TM	30%	TM
18,000×	TM	38%	21%	55%
54,000×	33%	TF	TF	TF
162,000×	TF	TF	TF	TF
486,000×	TF	TF	TF	TF

TM, too many >60% growth.

TF, too few <20% growth.

### Two-step library preparation for identification of cultivated bacteria by amplicon sequencing

We employed barcoded PCR to facilitate the characterization of our cultured microbe samples. However, we chose to develop a new barcoding approach relative to the published protocol by Zhang *et al*. due to a high cost of long primers required for the two-sided barcode approach utilized [34]. We developed an alternative strategy for barcode PCR that utilized two-step library preparation, which has been reported as having improved accuracy relative to one-step methods [36]. In two-step PCR, we were able to incorporate barcodes into the primary PCR primers for 16S rRNA gene amplification whilst still keeping the overall length relatively short and thus cost-effective (less than 60 bp). During this step, 96 wells from each plate get barcoded by the combination of 12 different forward and 8 different reverse primers described in Table S2. We chose to utilize primers that targeted the V4 region of the 16S rRNA gene for consistency with our routine microbiome workflows. The secondary PCR utilized the same primer sets as used for this method in routine microbiome sequencing [34], and the Illumina Nextera barcodes in secondary PCR primers barcoded each plate after combining primary barcoded wells from the first PCR into a single sample.

### Bioinformatic pipeline to analyze amplicon sequences from cultivated bacteria

To analyse multiplexed microbiome sequences from 96-well culture plates, we developed a pipeline in R using the DADA2 package [37], along with some custom functions for primer demultiplexing and sequence purity calculations. The pipeline is designed to be run as a command line script and performs four basic steps: (1) quality filtering, trimming and merging of raw forward and reverse sequence reads; (2) assignment of sequences to plate wells based on forward and reverse primer sequences; (3) counting and purity calculation of unique sequences with respect to their plate wells; and (4) taxonomy assignment of unique ASVs.

Purity is defined as the proportion of reads in a well that belong to a specific ASV, providing a measure of how dominant that ASV is in that well. A well with 100% purity means all reads belong to a single ASV, indicating a nearly pure culture, whereas lower purity suggests a mixed culture. This metric is useful for prioritizing wells most likely to yield the desired isolate upon streaking.

The output of the pipeline is a CSV file containing the ASV, the taxonomy and the top *n* ‘hits’ (i.e. plate wells, ranked by count) for each unique sequence. For each of the top *n* hits, the plate ID, well ID, sequence count and sequence purity are reported (Table 2).

**Table 2.** Example output of cultivated bacteria analysis pipeline

ASV	Sequence	Kingdom	Phylum	Class	Order	Family	Genus	top_count_1	top_count_2	top_count_3
14	AAGCGCGCGT…	Bacteria	Proteobacteria	Gammaproteobacteria	Pseudomonadales	Pseudomonadaceae	Pseudomonas	plate1_G2 | 1446 | 97.05	plate10_C6 | 997 | 100	plate16_D1 | 732 | 100
15	CGGTGAAATGC…	Bacteria	Proteobacteria	Gammaproteobacteria	Pseudomonadales	Pseudomonadaceae	Pseudomonas	plate33_H1 | 1343 | 96.55	plate33_H8 | 1269 | 98.99	plate30_B7 | 888 | 100
16	AGTATGGTAGT…	Bacteria	Proteobacteria	Gammaproteobacteria	Pseudomonadales	Pseudomonadaceae	Pseudomonas	plate25_E3 | 539 | 60.77	plate40_D12 | 466 | 100	plate31_E8 | 372 | 100
17	CGCGTAGGTGG…	Bacteria	Proteobacteria	Alphaproteobacteria	Rhizobiales	Rhizobiaceae	Neorhizobium	plate14_D5 | 2 | 100	plate1_A1 | 0 | NaN	plate1_A2 | 0 | 0
18	AACACCAGTGG…	Bacteria	Proteobacteria	Gammaproteobacteria	Pseudomonadales	Pseudomonadaceae	Pseudomonas	plate37_H8 | 2420 | 100	plate38_A6 | 1703 | 100	plate8_F2 | 703 | 100
19	AAAGCGTGGGG…	Bacteria	Proteobacteria	Gammaproteobacteria	Pseudomonadales	Pseudomonadaceae	Pseudomonas	plate16_D2 | 1230 | 98.72	plate38_E8 | 1070 | 100	plate37_A4 | 893 | 49.5
20	TACTGGGCGTA…	Bacteria	Proteobacteria	Gammaproteobacteria	Pseudomonadales	Pseudomonadaceae	Pseudomonas	plate21_D7 | 1350 | 92.85	plate25_F9 | 884 | 100	plate25_G6 | 808 | 100
21	GCGTGGGGAGC…	Bacteria	Proteobacteria	Gammaproteobacteria	Pseudomonadales	Pseudomonadaceae	Pseudomonas	plate38_F11 | 2432 | 97.51	plate4_E9 | 1859 | 98.1	plate12_B2 | 1483 | 100

For each unique ASV, the top wells (up to three) where it was most abundant are listed, including the plate ID, well ID, read count and calculated sequence purity (% of total reads in the well belonging to that ASV). This output is used to guide isolate recovery by identifying wells most likely to contain the target ASV in near-pure form.

### Comparisons of cultivated bacteria identity to the root slurry metagenome composition

**Fig. 1.**
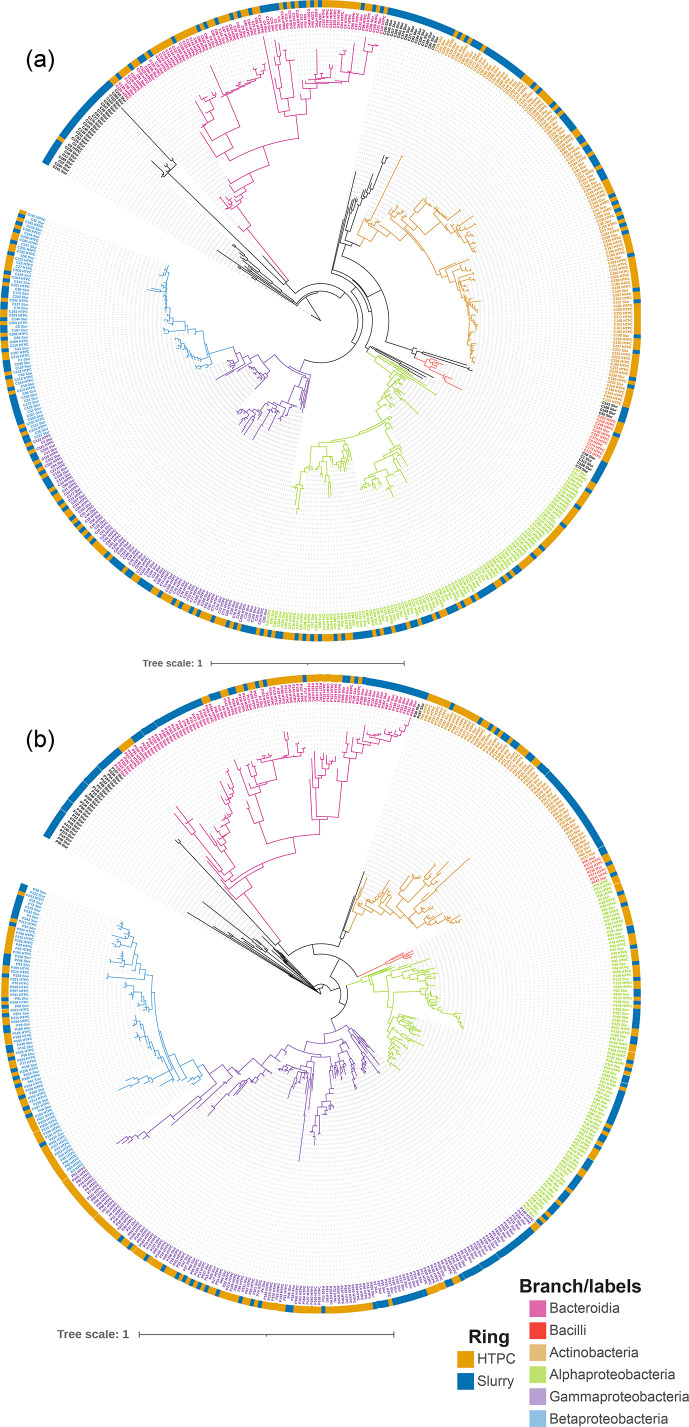
Maximum likelihood phylogenetic trees comparing the position of putatively culturable ASVs from the HTPC pipeline to the top 250 ASVs by abundance from the pea (**a**) and corn (**b**) microbiomes. Branches and labels of major lineages of bacteria from plant microbiomes are coloured (pink=Bacteroidia, red=Bacilli, brown=Actinobacteria, green=alpha proteobacteria, blue=beta proteobacteria and purple=gamma proteobacteria). The colour strip around the outside of the tree represents ASV origin, with HTPC ASVs in orange and microbiome ASVs in blue.

To investigate culture biases in our approaches, we generated phylogenetic trees from the ASVs identified in the HTPC pipeline (~250 each for pea and corn) and the top 250 ASVs identified by amplicon sequencing of the slurry microbiome used for culturing. Overall, we observed a widespread representation of groups of microbes that are associated with plant microbiomes including members of classes Bacteroidia, Bacilli, Actinobacteria, Alphaproteobacteria, Betaproteobacteria and Gammaproteobacteria. A more even distribution of HTPC and slurry ASVs is seen in the corn microbiome (Fig. 1), whereas the pea microbiome showed more evidence of bias. Less dominant classes with several representatives in the slurry but missing putatively culturable isolates included Saccharimonadia, Thermoleophilia and Acidmicrobiia. At the order level, most that had several representatives in the slurry included putatively culturable representatives in the pipeline. Orders with several representatives but without putatively culturable isolates included Micromonosporales, Pseudocardiales and Steroidobacterales.

To better understand the stable members of the root microbiome, we identified core ASVs within corn and pea roots using prevalence and abundance thresholds. Core ASVs were defined as those present in ≥70% of samples with a minimum relative abundance of 0.1%. Corn roots harboured the greatest number of core ASVs (86), whilst pea roots showed a smaller core (20 ASVs), and only 18 ASVs were shared across both hosts (Figs S1 and S2). Taxonomic classification of these ASVs revealed distinct patterns across host plants, with Proteobacteria dominating in corn and a more even representation of phyla such as Bacteroidota, Actinobacteria and Firmicutes observed in the pea core (Figs S1 and S2). These results suggest that whilst both crops share a small conserved microbial core, host-specific factors strongly influence the composition of stable microbial taxa associated with roots.

### Recovered isolates from pea and corn roots

**Fig. 2.**
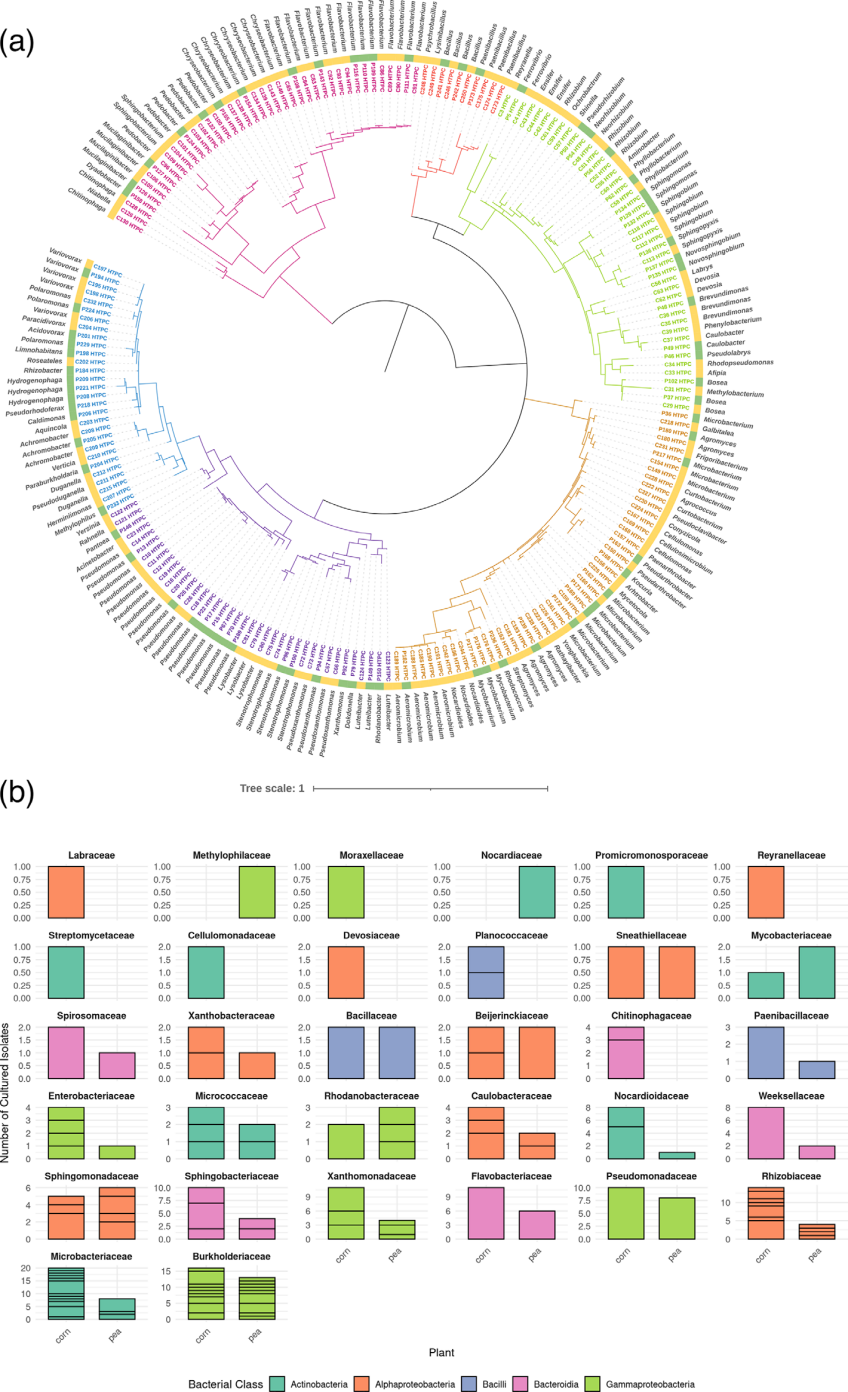
Taxonomy of successfully cultured and purified ASVs. (a) Maximum likelihood phylogenetic trees comparing the 215 cultured microbes from this work. Branches and labels of major lineages of bacteria from plant microbiomes are coloured (pink=Bacteroidia, red=Bacilli, brown=Actinobacteria, green=Alphaproteobacteria, blue=Betaproteobacteria and purple=Gammaproteobacteria). The colour strip around the outside of the tree represents ASV origin, with HTPC ASVs isolated from pea in green and ASVs isolated from corn in yellow. (**b) **Cultured ASVs grouped by plant host (corn and pea), faceted by bacterial family and coloured by class. Each bar represents the total number of cultured ASVs recovered from either corn or pea within each family. Horizontal lines within the bars indicate the number of unique genera represented within that family and plant host.

Based on the purity/number of reads, we selected ASVs to attempt to culture over 100 unique ASVs from each plant collection (pea and corn). The criteria for selection of attempted recovery were ASVs with ≥20% purity and ≥60 reads (121 out of 244 in pea; 200 out of 250 in corn). Following streak-plate purification of the isolates, they were authenticated by full-length 16S rRNA gene sequencing. We successfully recovered ~50% of the putatively culturable isolates (79 out of 121 in pea; 136 out of 200 in corn), with a slight bias towards success in the corn microbiome. We also noticed consistently that the number of colonies achieved during culture streaking approximately matched the relative number of reads for each isolate. The resulting isolates from pea and corn microbiomes showed similar phylogenetic distributions (Fig. 2a).

To assess how well-conserved members of the core microbiomes were captured through cultivation efforts, we examined the culturability and HTPC detection status of core ASVs using heatmaps stratified by host and taxonomy (Fig. S2). In the shared core microbiome, most genera exhibited low to moderate relative abundance, with a few, such as *Streptomyces*, *Pseudomonas* and *Pseudarthrobacter,* more consistently represented in culture-based datasets. In contrast, the corn-specific core microbiome displayed greater phylogenetic breadth, and several genera including *Rhizobium*, *Massilia* and *Variovorax* were detectable through either HTPC or isolate recovery. The pea-specific core microbiome was comparatively narrower and included more lineages that remain uncultured despite repeated attempts.

Interestingly, whilst only a few core ASVs were recovered directly, a larger proportion of their corresponding genera was successfully detected, underscoring the difficulty of recovering exact ASVs but the greater tractability of accessing broader taxonomic representatives. This was particularly evident in shared genera that included both cultured and uncultured ASVs, highlighting uneven culturability even within phylogenetically related groups. These patterns suggest that future culturing strategies may benefit from genus-level targeting, especially for functionally relevant but phylogenetically elusive taxa.

The recovered isolates represent a broad taxonomic range within the cultured collection, including members of Actinobacteria, Bacteroidota, Bacilli, Alphaproteobacteria and Gammaproteobacteria. Overall, corn roots yielded a greater diversity of cultured genera compared to pea, particularly within families such as *Microbacteriaceae*, *Burkholderiaceae* and *Flavobacteriaceae*. Some families, including *Sphingomonadaceae* and *Rhizobiaceae*, contributed cultured genera from both plant types, whilst others were host-specific (Fig. 2b). Fig. 2a, b illustrates the effectiveness of the culturing approach in recovering a wide array of bacterial taxa, including representatives from phyla typically considered difficult to isolate. Although this study does not yet report phenotypic profiling of isolates, the resulting culture collection is taxonomically resolved and sequence-linked to *in situ* community composition. This allows targeted future testing of isolates for traits such as plant growth promotion or stress tolerance. Moreover, the recovery of representatives from lineages like *Rhodococcus*, *Luteibacter* and *Streptomyces* highlights the potential for biotechnological utility in biocontrol or nutrient cycling. Such a collection represents a powerful tool to contrast synthetic communities derived from different hosts. All associated metadata, including Illumina V4 sequences (ASV_V4), HTPC detection, culturability status, full-length 16S rRNA sequences, blast-based taxonomic identification and classification into core, abundant or rare categories, are compiled in Table S3.

### Comparison to other methods

This method builds on advances in culturomics derived from the literature including colony picking, HTPC and cell sorting [34,35, 46, 47]. In specific, we adapted and optimized a protocol for high-throughput culturomics from the plant root microbiome which we applied to field-grown crop plants from ND, USA [34]. Based on our optimizations, we suggest increasing the dilution series for field plant-root samples over those previously recommended [34]. To decrease the cost of identification primers and increase accuracy, we employed a method involving a two-step barcoding PCR. Compared to similar protocols, our protocol does not require gel cutting, which decreases the cost, time and labour. It can also be deployed using a high-throughput library preparation protocol utilizing an acoustic liquid handling robot that we recently reported [38]. The described protocol employs the high-throughput identification of bacteria by sequencing the V4 region of the 16S rRNA gene. The V4 region of the 16S rRNA gene has been pointed out as the most reliable [48], reproducible [49], informative [50] and most similar to shotgun sequencing results [51] compared to other regions. We specifically chose the V4 region amplified by primers 515F/805R not only for its established use in large-scale microbiome studies (e.g. Earth Microbiome Project), but also because its shorter amplicon length (~291 bp) allows for higher-quality paired-end merging and more accurate ASV resolution. This was critical to ensure confident one-to-one matches between cultured isolate sequences and *in situ* community profiles.

In terms of bioinformatics, this protocol uses a pipeline built around the DADA2 package37 in R [52] to demultiplex the primers. DADA2 is one of the most used pipelines for processing amplicon sequencing data with great results and resolution [37]. Moreover, due to the versatility and open-source characteristics of R, several packages to analyse microbiome data have been developed [42,53,56]. This protocol uses the silva database [57] for taxonomy assignment of the sequenced reads, the recommended database in the DADA237 workflow. silva is the largest 16S rRNA gene dataset compared to RDP, Greengenes, NCBI and OTT [58].

## Conclusions

This study optimized and applied a high-throughput culturomics protocol to isolate and identify culturable bacterial taxa from the root microbiomes of field-grown pea and corn plants using NGS. The protocol combined dilution-to-extinction culturing with a two-step PCR and amplicon NGS strategy, successfully recovering around 50% of the unique putatively culturable amplicon ASVs identified through our unique bioinformatic pipeline. The resulting bacterial collection is taxonomically diverse, comprising plant-associated bacteria with potential applications in synthetic community design, evolutionary studies, comparative genomics, microbial ecology and microbiome research. The optimized protocol demonstrated enhanced accuracy, cost-effectiveness and high recovery rates, establishing a scalable and efficient method for large-scale culturing efforts in plant-microbiome research.

## Supplementary material

10.1099/mic.0.001571 Uncited Supplementary Material 1.

10.1099/mic.0.001571 Uncited Supplementary Material 2.

## Abbreviations

ASV: amplicon sequence variants
HTPC: high-throughput cultivation
NGS: next-generation sequencing
TSB: tryptic soy broth
