# Supplementary material for: Culturomics from field-grown crop plants using dilution to extinction, two-step library preparation and amplicon sequencing
Source: Microbiology (Reading). 2025 Jun 17;171(6):001571. doi: 10.1099/mic.0.001571 (PMC12174589; doi:10.1099/mic.0.001571)
Supplement: Uncited Supplementary Material 1. [file mic-171-01571-s001.pdf]

Supplementary Information

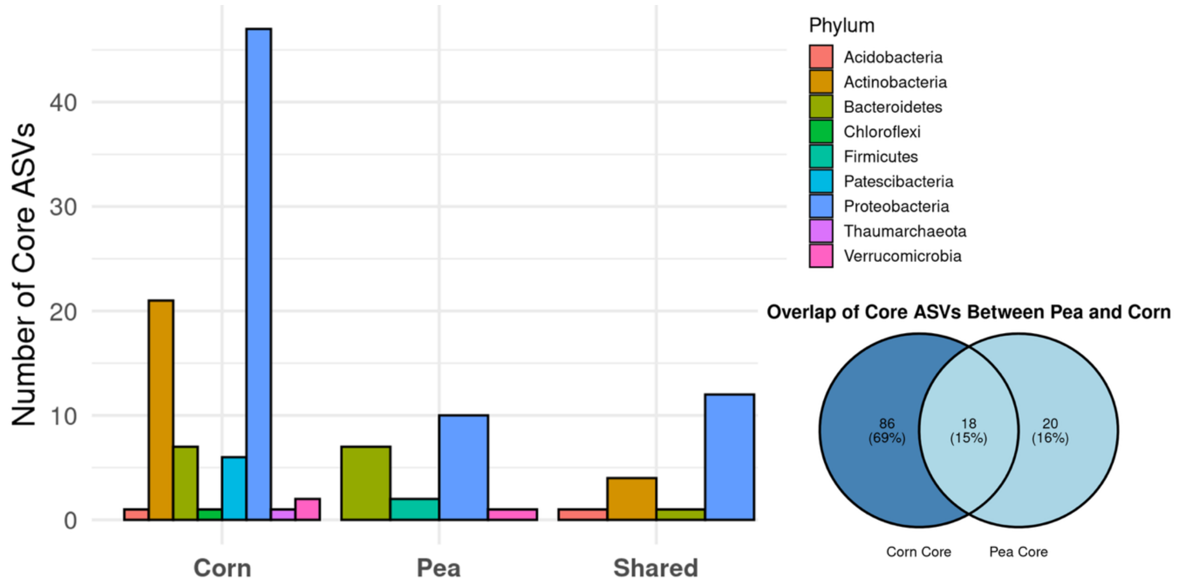

Supplementary Figure 1. Composition and Overlap of Core ASVs in Pea and Corn Root Microbiomes. (a) Bar plot showing the number of core ASVs detected in pea, corn, or both plant types, grouped by bacterial phylum. Core ASVs were defined as those present in at least 70% of samples within a plant type and exceeding a relative abundance threshold of 0.1%. Colors represent different phyla, highlighting taxonomic differences in the core communities across host plants. **(b)** Venn diagram illustrating the overlap of core ASVs between corn and pea. Of the total core ASVs, 86 were unique to corn, 20 unique to pea, and 18 were shared, indicating both conserved and host-specific microbial components in the root microbiomes of each crop.

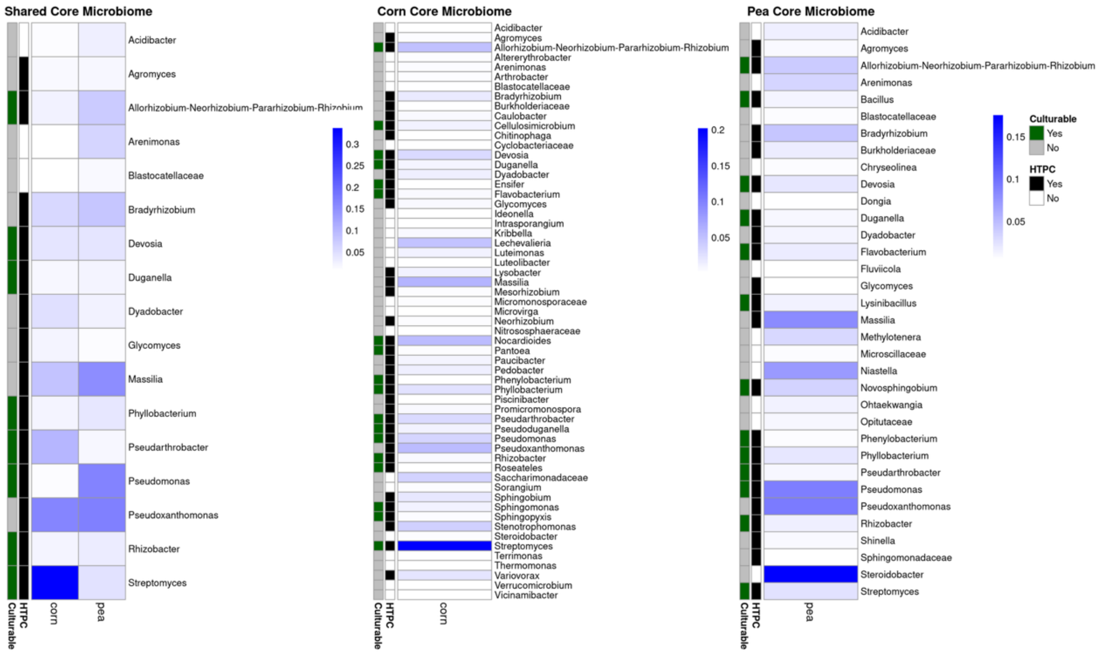

Supplementary Figure 2. Shared and Host-Specific Core Microbiomes in Corn and Pea Roots.

Heatmaps show the relative abundance of core ASVs (Amplicon Sequence Variants) across corn and pea samples, grouped by taxonomy (genus or family level). The left panel represents ASVs shared across both hosts, while the middle and right panels display ASVs unique to corn and pea core microbiomes, respectively. The intensity of blue indicates the mean relative abundance of each ASV. Rows are annotated with culturability status (green = cultured, gray = not cultured) and detection in high-throughput culturing (HTPC) pipeline (black = detected, white = not detected).

Supplementary Table 1. SRA accessions

BioSample	Bioproject	SRA	sample_name
SAMN46713596	PRJNA1220177	SRR32286237	Plate10_peadisease
SAMN46713597	PRJNA1220177	SRR32286236	Plate10_peasalinity
SAMN46713598	PRJNA1220177	SRR32286225	Plate1_peadisease
SAMN46713599	PRJNA1220177	SRR32286214	Plate1_peasalinity
SAMN46713600	PRJNA1220177	SRR32286203	Plate2_peadisease
SAMN46713601	PRJNA1220177	SRR32286192	Plate2_peasalinity
SAMN46713602	PRJNA1220177	SRR32286181	Plate3_peadisease
SAMN46713603	PRJNA1220177	SRR32286170	Plate3_peasalinity
SAMN46713604	PRJNA1220177	SRR32286167	Plate4_peadisease
SAMN46713605	PRJNA1220177	SRR32286166	Plate4_peasalinity
SAMN46713606	PRJNA1220177	SRR32286235	Plate5_peadisease
SAMN46713607	PRJNA1220177	SRR32286234	Plate5_peasalinity
SAMN46713608	PRJNA1220177	SRR32286233	Plate6_peadisease
SAMN46713609	PRJNA1220177	SRR32286232	Plate6_peasalinity
SAMN46713610	PRJNA1220177	SRR32286231	Plate7_peadisease
SAMN46713611	PRJNA1220177	SRR32286230	Plate7_peasalinity
SAMN46713612	PRJNA1220177	SRR32286229	Plate8_peadisease
SAMN46713613	PRJNA1220177	SRR32286228	Plate8_peasalinity
SAMN46713614	PRJNA1220177	SRR32286227	Plate9_peadisease

SAMN46713615	PRJNA1220177	SRR32286226	Plate9_peasalinity
SAMN46713616	PRJNA1220177	SRR32286224	corn_plate10_0nt
SAMN46713617	PRJNA1220177	SRR32286223	corn_plate11_0nt
SAMN46713618	PRJNA1220177	SRR32286222	corn_plate12_0nt
SAMN46713619	PRJNA1220177	SRR32286221	corn_plate13_0nt
SAMN46713620	PRJNA1220177	SRR32286220	corn_plate14_0nt
SAMN46713621	PRJNA1220177	SRR32286219	corn_plate15_0nt
SAMN46713622	PRJNA1220177	SRR32286218	corn_plate16_0nt
SAMN46713623	PRJNA1220177	SRR32286217	corn_plate17_0nt
SAMN46713624	PRJNA1220177	SRR32286216	corn_plate18_0nt
SAMN46713625	PRJNA1220177	SRR32286215	corn_plate19_0nt
SAMN46713626	PRJNA1220177	SRR32286213	corn_plate1_0nt
SAMN46713627	PRJNA1220177	SRR32286212	corn_plate20_0nt
SAMN46713628	PRJNA1220177	SRR32286211	corn_plate21_200nt
SAMN46713629	PRJNA1220177	SRR32286210	corn_plate22_200nt
SAMN46713630	PRJNA1220177	SRR32286209	corn_plate23_200nt
SAMN46713631	PRJNA1220177	SRR32286208	corn_plate24_200nt
SAMN46713632	PRJNA1220177	SRR32286207	corn_plate25_200nt
SAMN46713633	PRJNA1220177	SRR32286206	corn_plate26_200nt
SAMN46713634	PRJNA1220177	SRR32286205	corn_plate27_200nt
SAMN46713635	PRJNA1220177	SRR32286204	corn_plate28_200nt
SAMN46713636	PRJNA1220177	SRR32286202	corn_plate29_200nt

SAMN46713637	PRJNA1220177	SRR32286201	corn_plate2_0nt
SAMN46713638	PRJNA1220177	SRR32286200	corn_plate30_200nt
SAMN46713639	PRJNA1220177	SRR32286199	corn_plate31_200nt
SAMN46713640	PRJNA1220177	SRR32286198	corn_plate32_200nt
SAMN46713641	PRJNA1220177	SRR32286197	corn_plate33_200nt
SAMN46713642	PRJNA1220177	SRR32286196	corn_plate34_200nt
SAMN46713643	PRJNA1220177	SRR32286195	corn_plate35_200nt
SAMN46713644	PRJNA1220177	SRR32286194	corn_plate36_200nt
SAMN46713645	PRJNA1220177	SRR32286193	corn_plate37_200nt
SAMN46713646	PRJNA1220177	SRR32286191	corn_plate38_200nt
SAMN46713647	PRJNA1220177	SRR32286190	corn_plate39_200nt
SAMN46713648	PRJNA1220177	SRR32286189	corn_plate3_0nt
SAMN46713649	PRJNA1220177	SRR32286188	corn_plate40_200nt
SAMN46713650	PRJNA1220177	SRR32286187	corn_plate4_0nt
SAMN46713651	PRJNA1220177	SRR32286186	corn_plate5_0nt
SAMN46713652	PRJNA1220177	SRR32286185	corn_plate6_0nt
SAMN46713653	PRJNA1220177	SRR32286184	corn_plate7_0nt
SAMN46713654	PRJNA1220177	SRR32286183	corn_plate8_0nt
SAMN46713655	PRJNA1220177	SRR32286182	corn_plate9_0nt
SAMN46713656	PRJNA1220177	SRR32286180	slurry_corn0nt_r1
SAMN46713657	PRJNA1220177	SRR32286179	slurry_corn0nt_r2
SAMN46713658	PRJNA1220177	SRR32286178	slurry_corn0nt_r3

SAMN46713659	PRJNA1220177	SRR32286177	slurry_corn0nt_r4
SAMN46713660	PRJNA1220177	SRR32286176	slurry_corn200nt_r1
SAMN46713661	PRJNA1220177	SRR32286175	slurry_corn200nt_r2
SAMN46713662	PRJNA1220177	SRR32286174	slurry_corn200nt_r3
SAMN46713663	PRJNA1220177	SRR32286173	slurry_corn200nt_r4
SAMN46713664	PRJNA1220177	SRR32286172	slurry_peadisease_r1
SAMN46713665	PRJNA1220177	SRR32286171	slurry_peadisease_r2
SAMN46713666	PRJNA1220177	SRR32286169	slurry_peasalinity_r1
SAMN46713667	PRJNA1220177	SRR32286168	slurry_peasalinity_r2

Supplementary Table 2 . Primers used in this work

Primers for the well barcoding (HTPC PCR1)

Name	Sequence
V4_F_BC1	TCGTCGGCAGCGTCAGATGTGTATAAGAGACAGGCTAGTGCCAGCMGCCGCGGTAA
V4_F_BC2	TCGTCGGCAGCGTCAGATGTGTATAAGAGACAGTGTGTGTGCCAGCMGCCGCGGTAA
V4_F_BC3	TCGTCGGCAGCGTCAGATGTGTATAAGAGACAGAGTCTGGTGCCAGCMGCCGCGGTAA
V4_F_BC4	TCGTCGGCAGCGTCAGATGTGTATAAGAGACAGATCAGTGCCAGCMGCCGCGGTAA
V4_F_BC5	TCGTCGGCAGCGTCAGATGTGTATAAGAGACAGGACGAGTGCCAGCMGCCGCGGTAA
V4_F_BC6	TCGTCGGCAGCGTCAGATGTGTATAAGAGACAGTCGTCGGTGCCAGCMGCCGCGGTAA
V4_F_BC7	TCGTCGGCAGCGTCAGATGTGTATAAGAGACAGTGCTGTGCCAGCMGCCGCGGTAA
V4_F_BC8	TCGTCGGCAGCGTCAGATGTGTATAAGAGACAGCAGTTGTGCCAGCMGCCGCGGTAA
V4_F_BC9	TCGTCGGCAGCGTCAGATGTGTATAAGAGACAGACATGTGTGCCAGCMGCCGCGGTAA
V4_F_BC10	TCGTCGGCAGCGTCAGATGTGTATAAGAGACAGGCGGGTGCCAGCMGCCGCGGTAA
V4_F_BC11	TCGTCGGCAGCGTCAGATGTGTATAAGAGACAGGTTGAGTGCCAGCMGCCGCGGTAA
V4_F_BC12	TCGTCGGCAGCGTCAGATGTGTATAAGAGACAGGTGGCTGTGCCAGCMGCCGCGGTAA
V4_R_BC1	GTCTCGTGGGCTCGGAGATGTGTATAAGAGACAGGCTCACTACHVGGGTATCTAATCC
V4_R_BC2	GTCTCGTGGGCTCGGAGATGTGTATAAGAGACAGCTAGTACTACHVGGGTATCTAATCC
V4_R_BC3	GTCTCGTGGGCTCGGAGATGTGTATAAGAGACAGTAGATCACTACHVGGGTATCTAATCC
V4_R_BC4	GTCTCGTGGGCTCGGAGATGTGTATAAGAGACAGTCGCACTACHVGGGTATCTAATCC
V4_R_BC5	GTCTCGTGGGCTCGGAGATGTGTATAAGAGACAGCCTTAACTACHVGGGTATCTAATCC
V4_R_BC6	GTCTCGTGGGCTCGGAGATGTGTATAAGAGACAGCATAACACTACHVGGGTATCTAATCC

V4_R_BC7	GTCTCGTGGGCTCGGAGATGTGTATAAGAGACAGCAGAACTACHVGGGTATCTAATCC
V4_R_BC8	GTCTCGTGGGCTCGGAGATGTGTATAAGAGACAGTGTTCACTACHVGGGTATCTAATCC

Primers for the plate barcoding (HTPC PCR2)

Name	Sequence
F1_MetaIndex	AATGATACGGCGACCACCGAGATCTACACTATAGCCTTCGTCGGCAGCGTC
F2_MetaIndex	AATGATACGGCGACCACCGAGATCTACACATAGAGGCTCGTCGGCAGCGTC
F3_MetaIndex	AATGATACGGCGACCACCGAGATCTACACCCTATCCTTCGTCGGCAGCGTC
F4_MetaIndex	AATGATACGGCGACCACCGAGATCTACACGGCTCTGATCGTCGGCAGCGTC
F5_MetaIndex	AATGATACGGCGACCACCGAGATCTACACAGGCGAAGTCGTCGGCAGCGTC
F6_MetaIndex	AATGATACGGCGACCACCGAGATCTACACTAATCTTATCGTCGGCAGCGTC
F7_MetaIndex	AATGATACGGCGACCACCGAGATCTACACCAGGACGTTCGTCGGCAGCGTC
F8_MetaIndex	AATGATACGGCGACCACCGAGATCTACACGTACTGACTCGTCGGCAGCGTC
F9_MetaIndex	AATGATACGGCGACCACCGAGATCTACACTGAACCTTTCGTCGGCAGCGTC
F10_MetaIndex	AATGATACGGCGACCACCGAGATCTACACTAGATCGCTCGTCGGCAGCGTC
F11_MetaIndex	AATGATACGGCGACCACCGAGATCTACACCTCTCTATTCGTCGGCAGCGTC
F12_MetaIndex	AATGATACGGCGACCACCGAGATCTACACTATCCTCTTCGTCGGCAGCGTC
F13_MetaIndex	AATGATACGGCGACCACCGAGATCTACACAGAGTAGATCGTCGGCAGCGTC
F14_MetaIndex	AATGATACGGCGACCACCGAGATCTACACGTAAGGAGTCGTCGGCAGCGTC
F15_MetaIndex	AATGATACGGCGACCACCGAGATCTACACACTGCATATCGTCGGCAGCGTC
F16_MetaIndex	AATGATACGGCGACCACCGAGATCTACACAAGGAGTATCGTCGGCAGCGTC
R13_MetaIndex	CAAGCAGAAGACGGCATACGAGATGTCGTGATGTCTCGTGGGCTCGG

R14_MetaIndex	CAAGCAGAAGACGGCATACGAGATCGAGTAATGTCTCGTGGGCTCGG
R15_MetaIndex	CAAGCAGAAGACGGCATACGAGATTCTCCGGAGTCTCGTGGGCTCGG
R16_MetaIndex	CAAGCAGAAGACGGCATACGAGATAATGAGCGGTCTCGTGGGCTCGG
R17_MetaIndex	CAAGCAGAAGACGGCATACGAGATGGAATCTCGTCTCGTGGGCTCGG
R18_MetaIndex	CAAGCAGAAGACGGCATACGAGATTTCTGAATGTCTCGTGGGCTCGG
R19_MetaIndex	CAAGCAGAAGACGGCATACGAGATACGAATTCGTCTCGTGGGCTCGG
R20_MetaIndex	CAAGCAGAAGACGGCATACGAGATAGCTTCAGGTCTCGTGGGCTCGG
R21_MetaIndex	CAAGCAGAAGACGGCATACGAGATGCGCATTAGTCTCGTGGGCTCGG
R22_MetaIndex	CAAGCAGAAGACGGCATACGAGATCATAGCCGGTCTCGTGGGCTCGG
R23_MetaIndex	CAAGCAGAAGACGGCATACGAGATTTCGCGGAGTCTCGTGGGCTCGG
R24_MetaIndex	CAAGCAGAAGACGGCATACGAGATGCGCGAGAGTCTCGTGGGCTCGG
R1_MetaIndex	CAAGCAGAAGACGGCATACGAGATTCGCCTTAGTCTCGTGGGCTCGG
R2_MetaIndex	CAAGCAGAAGACGGCATACGAGATCTAGTACGGTCTCGTGGGCTCGG
R3_MetaIndex	CAAGCAGAAGACGGCATACGAGATTTCTGCCTGTCTCGTGGGCTCGG
R4_MetaIndex	CAAGCAGAAGACGGCATACGAGATGCTCAGGAGTCTCGTGGGCTCGG
R5_MetaIndex	CAAGCAGAAGACGGCATACGAGATAGGAGTCCGTCTCGTGGGCTCGG
R6_MetaIndex	CAAGCAGAAGACGGCATACGAGATCATGCCTAGTCTCGTGGGCTCGG
R7_MetaIndex	CAAGCAGAAGACGGCATACGAGATGTAGAGAGGTCTCGTGGGCTCGG
R8_MetaIndex	CAAGCAGAAGACGGCATACGAGATCCTCTCTGGTCTCGTGGGCTCGG
R25_MetaIndex	CAAGCAGAAGACGGCATACGAGATCTATCGCTGTCTCGTGGGCTCGG
R10_MetaIndex	CAAGCAGAAGACGGCATACGAGATCAGCCTCGGTCTCGTGGGCTCGG
R11_MetaIndex	CAAGCAGAAGACGGCATACGAGATTGCCTCTTGTCTCGTGGGCTCGG

R12_MetaIndex	CAAGCAGAAGACGGCATACGAGATTCCTCTACGTCTCGTGGGCTCGG

Primers for sequencing the slurry microbiome

Name	Sequence
V4_515F	TCGTCGGCAGCGTCAGATGTGTATAAGAGACAGGTGCCAGCMGCCGCGGTAA
V4_ 805R	GTCTCGTGGGCTCGGAGATGTGTATAAGAGACAGGACTACHVGGGTATCTAATCC

Primers for full-length 16S rRNA gene amplification

Name	Sequence
27F	AGAGTTTGATCCTGGCTCAG
1492R	TACGGCTACCTTGTTACGACTT

Supplementary Data 3 . Metadata and ecological classification of cultured and non-cultured ASVs from pea and corn root microbiomes is available as a supplemental excel file.
